# The metabolic syndrome- associated small G protein ARL15 plays a role in adipocyte differentiation and adiponectin secretion

**DOI:** 10.1038/s41598-017-17746-8

**Published:** 2017-12-14

**Authors:** Nuno Rocha, Felicity Payne, Isabel Huang-Doran, Alison Sleigh, Katherine Fawcett, Claire Adams, Anna Stears, Vladimir Saudek, Stephen O’Rahilly, Inês Barroso, Robert K. Semple

**Affiliations:** 1 The University of Cambridge Metabolic Research Laboratories, Wellcome Trust-MRC Institute of Metabolic Science, Cambridge, UK; 2 The National Institute for Health Research Cambridge Biomedical Research Centre, Cambridge, UK; 3 Wolfson Brain Imaging Centre, University of Cambridge School of Clinical Medicine, Cambridge Biomedical Campus, Cambridge, UK; 4 National Institute for Health Research/Wellcome Trust Clinical Research Facility, Cambridge University Hospitals NHS Foundation Trust, Cambridge Biomedical Campus, Cambridge, UK; 5 Wellcome Trust Sanger Institute, Wellcome Genome Campus, Hinxton, UK; 6 Wolfson Diabetes and Endocrine Clinic, Institute of Metabolic Science, Addenbrooke’s Hospital, Cambridge University Hospitals NHS Foundation Trust, Cambridge, UK; 7 Centre for Cardiovascular Sciences, University of Edinburgh, Queen’s Medical Research Institute, Edinburgh, UK

## Abstract

Common genetic variants at the *ARL15* locus are associated with plasma adiponectin, insulin and HDL cholesterol concentrations, obesity, and coronary atherosclerosis. The *ARL15* gene encodes a small GTP-binding protein whose function is currently unknown. In this study adipocyte-autonomous roles for ARL15 were investigated using conditional knockdown of *Arl15* in murine 3T3-L1 (pre)adipocytes. *Arl15* knockdown in differentiated adipocytes impaired adiponectin secretion but not adipsin secretion or insulin action, while in preadipocytes it impaired adipogenesis. In differentiated adipocytes GFP-tagged ARL15 localized predominantly to the Golgi with lower levels detected at the plasma membrane and intracellular vesicles, suggesting involvement in intracellular trafficking. Sequencing of *ARL15* in 375 severely insulin resistant patients identified four rare heterozygous variants, including an early nonsense mutation in a proband with femorogluteal lipodystrophy and non classical congenital adrenal hyperplasia, and an essential splice site mutation in a proband with partial lipodystrophy and a history of childhood yolk sac tumour. No nonsense or essential splice site mutations were found in 2,479 controls, while five such variants were found in the ExAC database. These findings provide evidence that ARL15 plays a role in adipocyte differentiation and adiponectin secretion, and raise the possibility that human ARL15 haploinsufficiency predisposes to lipodystrophy.

## Introduction

Several metabolic traits, including insulin resistance and hyperinsulinemia, low HDL cholesterol, and hypoadiponectinemia, are strongly associated with each other, and with major diseases including diabetes mellitus and atherosclerosis. However, unpicking the direction of causality in such associations is a major challenge in which genetic studies play an important role. Genome-wide association studies (GWAS) agnostically seek statistical associations among common genetic variation and traits of interest. If the pertinent change in gene(s) expression or function that drives such genetic association can be identified, which usually requires fine mapping of the initial GWAS signal, then insights into disease mechanism may in principle be garnered. However most genetic signals identified by GWAS do not lie in protein-coding regions of genes, and uncertainty about the protein or transcript-level perturbation directly associated with the trait of interest presents a bottleneck to full realisation of the power of GWAS as a tool to shed light on disease mechanism.

This study set out to investigate *ARL15*, a gene that encodes a small GTP binding protein of unknown function, and that has been implicated in metabolic traits and diseases by several different GWAS and other population-wide genetic studies. Genetic variation at a locus including only *ARL15* was first associated with HDL cholesterol concentration and also plasma concentration of adiponectin^1^, a highly abundant and stable plasma protein almost exclusively secreted by adipocytes, and widely believed to act as an insulin-sensitising adipokine^2^. The same common genetic variants at the *ARL15* locus are associated with indices of insulin resistance and coronary heart disease^1^, with other studies confirming variation at the *ARL15* locus to be associated with fasting insulin, HDL cholesterol and triglyceride concentrations^3–5^.

Based on similarities between the pattern of metabolic traits associated with *ARL15* locus variants, and the pattern of metabolic derangement seen in Mendelian lipodystrophies, it has been hypothesized that the genetic variation at the *ARL15* locus may affect metabolic traits such as fasting insulin and HDL concentrations through a primary action on adipose tissue development or function^6,7^. Indeed, SNPs at the *ARL15* locus have been associated with body shape^8^ and waist circumference^9^, and increased gene dosage has been associated with childhood obesity^10^.

We thus set out firstly to assess whether ARL15 plays a cell autonomous role in the biosynthesis or secretion of adiponectin and/or preadipocyte differentiation using conditional Arl15 knockdown in the insulin-responsive, adiponectin-producing 3T3-L1 cell line, and secondly, to seek rare coding sequence variants in *ARL15* in a cohort of volunteers with lipodystrophic or non lipodystrophic severe insulin resistance.

## Methods

All human studies were approved by the National Health Service Research Ethics Committee of the United Kingdom and were conducted in accordance with the principles of the Declaration of Helsinki. Participants gave informed consent both for study participation and for use of clinical images where relevant.

### Antibodies and Immunoassays

Antibodies are listed in Supplementary Table 1. The mouse adipsin ELISA kit was from Millipore. The murine adiponectin autoDELFIA immunoassay was previously described^11^. Rabbit polyclonal antibody to adiponectin used in immunofluorescence experiments was a gift from Dr. P.E. Scherer.

### Cell culture

Reagents were from SIGMA unless otherwise indicated. Cells were maintained in DMEM with 10% neonatal calf serum (3T3-L1s) or fetal bovine serum (FBS) (HEK293T). To induce adipocyte differentiation, 2-day postconfluent 3T3-L1 preadipocytes were transferred to DMEM-Tet-Approved FBS (Clontech), supplemented with 500 µmol/L IBMX, 1 µmol/L dexamethasone, 10 µmol/L insulin, and 100 nmol/L rosiglitazone for 3 days. Cells were further cultured in DMEM supplemented with 10% Tet-Approved FBS, 10 µmol/L insulin (Actrapid, Novo Nordisk) for 3 days and thereafter in DMEM with Tet-Approved FBS for 8 days unless otherwise indicated. Differentiation was assessed by Oil-Red-O staining^12^.

### Plasmid construction

Cloning of murine *Arl15* into the mammalian pEGFP-N3 and the lentiviral pSLIK vectors, and shArl15 pSLIK is described in detail in Supplementary Information. The CFP-tagged fusions of the Golgin-245/Furin Golgi markers have been described previously^13^.

### Generation of Stable Cell Lines Conditionally Expressing shArl15

Lentiviral particles were generated as previously described^14^. Low-passage 3T3-L1 preadipocytes were transduced at MOI ≤1 in the presence of polybrene and selected in G418. For RNAi experiments clones were selected using cloning cylinders. Clones with optimal RNAi inducibility, determined by GFP fluorescence and RT-qPCR, were pooled prior to differentiation. For subcellular localization studies, polyclonal 3T3-L1 lines with doxycycline-inducible Arl15-GFP expression were generated by G418 selection prior to adipocyte differentiation. All pSLIK-transduced cell lines were maintained in G418-supplemented DMEM-NCS, with antibiotics withdrawn for adipocyte differentiation and doxycycline induction. The pSLIK lentiviral system has been described previously^15^.

### Gene Expression Analysis

Total RNA extracted with RNeasy Mini Kit (Qiagen) was used for MMLV reverse transcription (Promega). RT-qPCR used either custom designed or ABI TaqMan Gene Expression Assays run with ABI TaqMan Mastermix and analyzed on ABI 7900 HT system. Primer/probes were designed using Primer-Express (ABI). Catalog numbers and primer/probe sequences are listed in Supplementary Table 2.

For immunoblotting, adipocytes were washed twice in ice-cold PBS and lysed on ice in 50 mmol/L HEPES, pH 7.5, 150 mmol/L NaCl, 30 mmol/L NaF, 10 mmol/L Na_4_P_2_O_7_, 1 mmol/L Na_2_VO_4_, 1% Triton X-100, 10 mmol/L EDTA and Protease Complete Cocktail (Roche). Extracts were cleared by centrifugation and protein concentrations determined using Bio-Rad DC Protein Assay. Proteins were mixed with sample buffer (3% SDS, 50 mM Tris-HCl pH 6.8, 5% 2-mercaptoethanol, 12% glycerol) and heat-denatured at 95 °C. For complete reduction of adiponectin samples this buffer was supplemented with 10 mM dithiothreitol. For non-reducing non-heat-denaturing conditions, samples were incubated with reducing agent-free sample buffer for 1 h at room temperature. Proteins were resolved by SDS-PAGE and transferred to nitrocellulose membranes using iBlot (Invitrogen). Membranes were blocked in 5% (w/v) skimmed milk or 3% (w/v) BSA (for phospho-protein immunodetection) TBS-T.

### Analysis of adiponectin from lysates and medium of 3T3-L1 adipocytes and human serum

3T3-L1 adipocytes were washed twice with PBS and transferred to serum-free medium containing 3% BSA for 24 h. Medium (10 μL) was harvested, centrifuged and analyzed by immunoblotting under non-reducing and non-heat-denaturing or reducing/denaturing conditions. For analysis of adiponectin in lysates, protein samples were obtained as described for immunoblotting of cell lysates. For analysis of human serum adiponectin, serum samples were diluted 1:50 and processed as described above for adipocyte culture medium. Adipocyte medium samples were diluted 1:50 for adiponectin DELFIA analysis and 1:10 for adipsin ELISA. Human serum samples were diluted 1:50 for adiponectin DELFIA.

### Deoxyglucose uptake assay

Insulin-stimulated deoxyglucose uptake was assayed as previously described^16^. Briefly, DOX-treated and serum-starved adipocytes were insulin-stimulated and incubated with [^3^H]deoxyglucose, washed and lysed. Radioactivity was normalized to protein concentration.

### Immunofluorescence microscopy

For detailed methods on (pre)-adipocytes and NMuMG immunofluorescence studies see Supplementary Information.

### Genetic studies

*ARL15* (NM_019087.2) was screened in a total of 375 individuals ascertained by severe insulin resistance and 2,479 controls, using several different sequencing approaches (see Supplementary Information for details of cohorts studied and sequencing techniques). All rare *ARL15* alleles reported were verified in non-amplified genomic DNA using Sanger sequencing. For assessment of *ARL15* exon 4 splicing from whole-blood RNA see Supplementary Information.

### Human imaging and physiological studies

Body composition was assessed using Lunar Prodigy dual-energy X-ray absorptiometry (DEXA, GE Lunar). Hepatic triglyceride was assessed either using proton magnetic resonance spectroscopy on a Siemens 3T-Verio scanner^17^ (Patient 1) or using MRI proton density fat fractionation with IDEAL-IQ^18^ (Patient 2). Adipose distribution was determined by magnetic resonance imaging, using T1-weighted turbo spin echo, water-suppressed, transaxial images.

Insulin, leptin and adiponectin were assayed using two-step time-resolved AutoDELFIA immunoassays^11^. Normative adiponectin, leptin and oral glucose tolerance testing data were derived from the MRC Ely Study cohort^19^. All other biochemical analyses were undertaken in an accredited clinical diagnostic laboratory in Addenbrooke’s Hospital, Cambridge, UK.

## Results

### Arl15 knockdown impairs adiponectin expression and secretion

**Figure 1:**
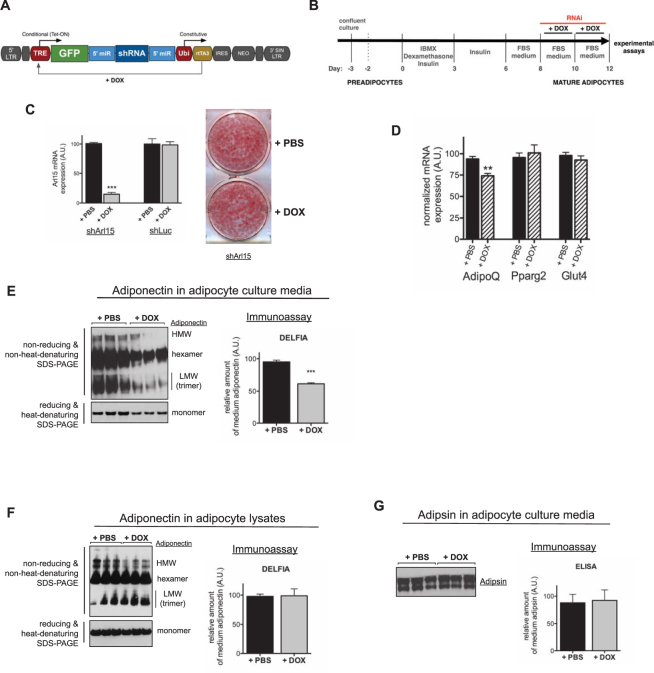
Arl15 knockdown impairs adiponectin, but not adipsin, secretion from 3T3-L1 adipocytes. (**A**) schematic of the pSLIK lentiviral vector used for doxycycline (DOX)-induced expression of a short hairpin RNA targeting Arl15 (shArl15). (**B**) Protocols for adipocyte differentiation and doxycycline (DOX)-induction of RNAi in 3T3-L1 adipocytes. (**C**) DOX-induced suppression of Arl15 by RNAi in adipocytes. Left, Arl15 mRNA expression in shArl15 adipocytes, or adipocytes conditionally expressing shRNA against firefly luciferase (shLuc), treated with DOX or PBS. The same conditions were used in. Right, representative image of Oil-Red-O stained lipid droplets in shArl15 3T3-L1 adipocytes treated with DOX (RNAi) or PBS (control), as indicated. (**D**) Adiponectin (AdipoQ), peroxisome proliferator-activated receptor gamma-2 (Pparg2), and Glut4 mRNA expression in shArl15 adipocytes treated with PBS (black bars) or DOX (hatched bars) determined by real-time quantitative PCR and normalised to murine CypA (PBS, *n* = 3; DOX, *n* = 3). Data are expressed as mean ± SEM; differences between means analyzed by Student’s *t* test; ***P > 0.001. (**E**) Total adiponectin (bottom left) or its oligomeric profile (top left) in shArl15 adipocyte culture medium assessed by SDS-PAGE under reducing, heat-denaturing or non-reducing, non-heat-denaturing SDS-PAGE respectively prior to immunoblotting. HMW (high-molecular weight, ≥9mer), hexamer, LMW (low molecular weight, trimer), and monomer are indicated. Right panel, quantification adiponectin concentrations in media from DOX- or PBS-treated shArl15 cultures measured by DELFIA immunoassay. (**F**) Left panel, total adiponectin (lower immunoblot) and its oligomeric profile (top immunoblot) in lysates of shArl15 adipocytes treated with DOX or PBS (control) revealed by Western blotting. Right panel, quantification of intracellular adiponectin determined by immunoassay (DELFIA). (**G**) Left panel, immunoblotting of adipsin in shArl15 adipocyte medium. Right panel, relative amounts of adipsin in culture medium determined using a mouse adipsin enzyme-linked immunoassay (ELISA). Means ± SEM are shown (*n* = 3).

The murine 3T3-L1 preadipocyte cell line can be differentiated with high efficiency into lipid-laden adipocytes that exhibit many characteristics of primary adipocytes, including adiponectin secretion and insulin-stimulated glucose uptake. To investigate cell-autonomous consequences of Arl15 deficiency in mature adipocytes without potential confounding by any effect of the knockdown on differentiation, 3T3-L1 adipocytes conditionally expressing microRNA-like shRNA targeting murine *Arl15* (shArl15) under the control of a doxycycline (DOX)-responsive promoter were generated (Fig. 1A–C). DOX-induced RNAi acutely reduced *Arl15* transcript levels by >75% in differentiated adipocytes relative to non-induced shArl15 adipocytes, with no discernible effect on adipocyte differentiation, as assessed by Oil-Red-O staining of lipid droplets (Fig. 1C). Acute knockdown of *Arl15* moderately but significantly reduced *AdipoQ* mRNA levels, but had no effect on the mRNA of peroxisome proliferator-activated receptor gamma-2 (*Pparg2*) or *Glut4* (Fig. 1D). *Arl15* knockdown reduced total adiponectin secretion into cell culture medium, as assessed by either immunoblotting or immunoassay (Fig. 1E), and immunoblotting under non-reducing, non-heat-denaturing conditions revealed that all oligomeric forms of secreted adiponectin were affected (Fig. 1E). Despite the reduction in *AdipoQ* mRNA and secreted adiponectin, intracellular adiponectin levels were not affected by *Arl15* knockdown, however (Fig. 1F).

Given the effect of Arl15 downregulation on adiponectin secretion and the key role that some ARF and ARL (ARF-like) small G proteins have in intracellular transport pathways^20–23^, we assessed whether Arl15 suppression caused generalised inhibition of intracellular protein trafficking and secretion. However we found that secretion of adipsin, an adipokine whose trafficking route to the cell surface of 3T3-L1 adipocytes partly overlaps with that of adiponectin^24^, was not affected by suppression of Arl15, as assessed by either immunoblotting or ELISA (Fig. 1G).

### Arl15 knockdown has no impact on adipocyte-autonomous insulin action

**Figure 2:**
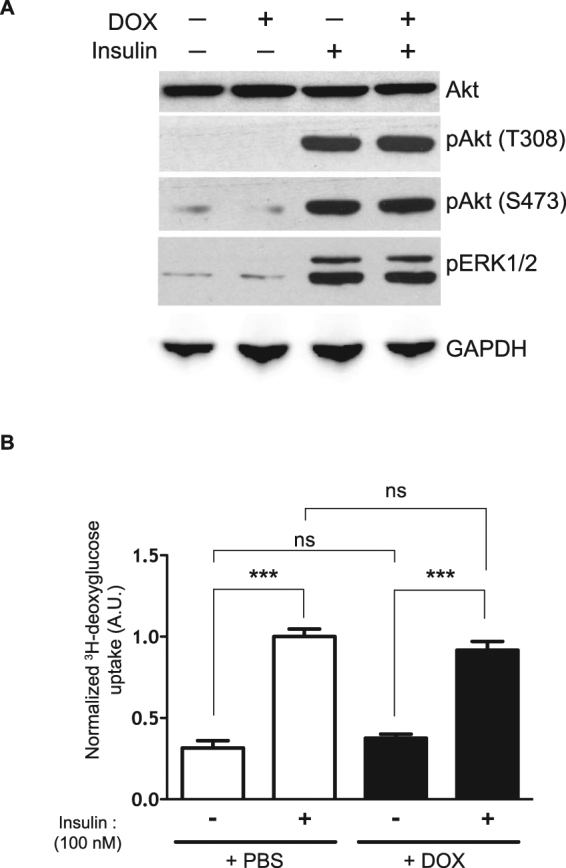
Arl15 knockdown has no impact on adipocyte-autonomous insulin action. (**A**) Insulin-stimulated phosphorylation of Akt on Threonine 308 (pAkt (T308) and Serine 473 (pAKT (S473)) or extracellular signal-regulated kinase 1/2 (pERK1/2) after 5-min treatment of serum-starved shArl15 adipocytes with 100 nmol/L insulin, assessed by Western blotting with GAPDH (GAPDH) and Akt shown as a loading controls (Akt). Arl15 knockdown was induced by DOX treatment. (**B**) Relative [^3^H]deoxyglucose uptake by Arl15 knockdown (+DOX) or control (+PBS) serum-starved shArl15 adipocytes treated with 100 nmol/L insulin or saline for 30 min. Means ± SEM are shown (*n* = 6); A.U., arbitrary units. Significance by ANOVA followed by Bonferroni Multiple Comparison tests was set at *P* < 0.05. ns = *P* > 0.05; ***P < 0.001.

We next tested whether Arl15 is involved in insulin signal transduction and/or insulin-responsive glucose uptake. Arl15 knockdown had no effect on peak insulin-stimulated phosphorylation of either AKT or ERK (extracellular signal-regulated kinase) (Fig. 2A), or on basal or insulin-stimulated glucose uptake (Fig. 2B), suggesting that Arl15 is not involved in insulin-stimulated mobilisation and fusion of Glut4-containing vesicles with the plasma membrane.

### Arl15 knockdown impairs 3T3-L1 pre-adipocyte differentiation

**Figure 3:**
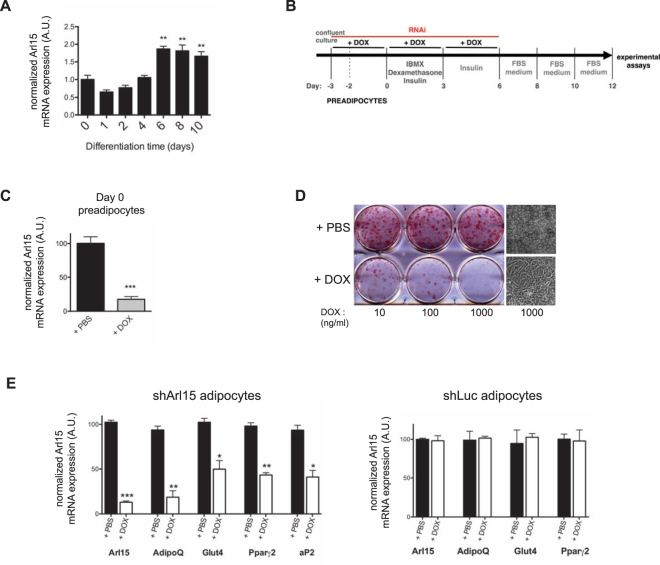
Arl15 knockdown impairs 3T3-L1 adipocyte differentiation. (**A**) Arl15 mRNA expression during differentiation of wild-type 3T3-L1 adipocytes, determined by real-time quantitative PCR. **P < 0.01 vs. day 0 (*n* = 4). (**B**) DOX-induced RNAi and differentiation protocols used to assess the effects of Arl15 knockdown on 3T3-L1 adipocyte differentiation. (**C**) Arl15 mRNA expression in 3T3-L1 preadipocytes harvested at day 0 of the RNAi and differentiation protocol described above. (**D**) Oil-Red-O staining of triglycerides in shArl15 adipocytes treated with either increasing concentrations of doxycycline (+DOX) or saline solution (+PBS), as indicated. Far right, representative bright field micrographs of shArl15 adipocytes treated with PBS (top) or 1 mg/mL DOX (bottom). (**E**) Arl15, adiponectin (AdipoQ), peroxisome proliferator-activated receptor gamma-2 (Pparg2), Glut4, and adipocyte protein 2 (aP2) mRNA expression in shArl15 adipocytes treated with DOX (hatched) or PBS (black), determined by real-time quantitative PCR. The same experiment was repeated using shLuc adipocytes, as control (chart on the right). mRNA levels were normalized to those of mouse CypA (cyclophilin A) (PBS, *n* = 3; DOX, *n* = 3). All data are shown as means ± SEM. A.U., arbitrary units. Differences between means were analysed by Student’s *t* test; *P < 0.05, **P < 0.01, ***P < 0.001.

Most monogenic insulin resistance is caused not by direct perturbation of cell autonomous insulin signalling, but by defects in adipocyte differentiation or function. Given the pattern of associations of the common *ARL15* locus variant, which are suggestive of adipose dysfunction, a role of ARl15 in adipocyte differentiation was next assessed. Expression of Arl15 mRNA was significantly increased by day 6 of 3T3-L1 adipocyte differentiation and remained unchanged until day 10 (Fig. 3A). Knockdown of Arl15 prior to adipocyte differentiation was then undertaken (Fig. 3B). Arl15 mRNA expression at day 0 in knockdown cells was reduced to < 25% that of shArl15 3T3-L1s treated with saline (Fig. 3C), and the vast majority of DOX-treated cells failed to acquire the characteristic morphological features of differentiated adipocytes, including accumulation of cytoplasmic lipid droplets. Adipocyte differentiation was highly sensitive to Arl15 knockdown, with impaired lipid droplet formation seen at DOX concentrations as low as 10 ng/mL (Fig. 3D). Reduced transcript levels of adipocyte markers further corroborated impaired differentiation of Arl15 knockdown cells (Fig. 3E).

### Arl15 predominantly localizes to the Golgi complex

**Figure 4:**
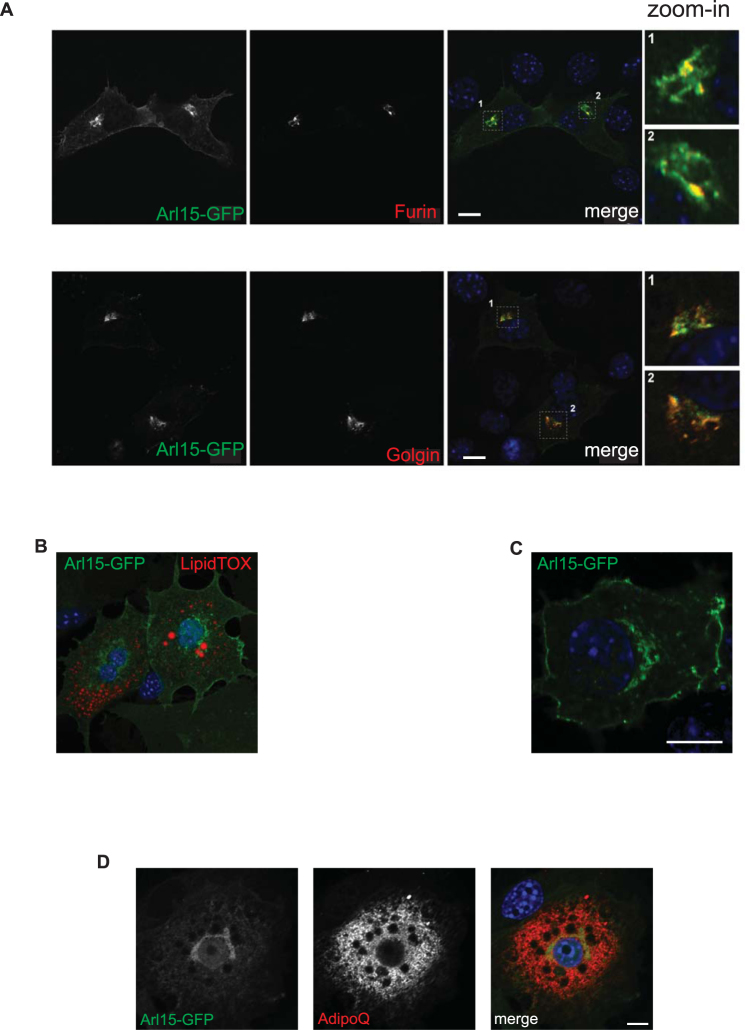
Arl15 predominantly localizes to the Golgi complex. (**A**) Wild-type 3T3-L1 preadipocytes were co-transfected with GFP-tagged Arl15 and either CFP-Golgin or CFP-Furin, two Golgi markers, fixed, and imaged by confocal microscopy. Arl15-GFP and CFP-Furin (top) or CFP-Golgin (bottom) are show in grayscale and overlayed on the right (merge) in green and red, respectively. Insets on the far right (zoom-in) show in detail regions 1 or 2, as indicated in the corresponding merge panel. (**B**) Day-3 3T3-L1 adipocytes expressing GFP-tagged Arl15 (green), fixed, and stained with neutral lipid stain LipidTOX (red) and DAPI (blue). A representative confocal micrograph of adipocytes expressing Arl15-GFP at the perinuclear region, intracellular vesicles, and the plasma membrane. (**C**) NMuMG cell transiently expressing Arl15-GFP (green) at the plasma membrane, internal vesicles, and the perinuclear region. (**D**) Day-5 3T3-L1 adipocytes expressing GFP-tagged Arl15 (green) were fixed and immunostained with anti-adiponectin antibodies and Alexa Fluor 594 (AdipoQ, red). Panel on the right shows the two channels merged. The average Pearson’s correlation coefficient (*r*) for the GFP and Alexa channels in hand-drawn “regions of interest” (ROI) encompassing entire cells was: 0.39 (without Costes automatic thresholding), 0.02 (below Costes Auto thresholding), and 0.11 (above Costes automatic thresholding). Scale bars, 10 μm.

To investigate the subcellular localization of Arl15, GFP-tagged Arl15 was expressed in 3T3-L1 cells and visualised by fluorescence confocal microscopy. Arl15-GFP was found in a predominantly perinuclear distribution characteristic of the Golgi apparatus, with some signal also in intracellular vesicles. 3T3-L1 preadipocytes were then co-transfected with Arl15-GFP and two CFP-tagged Golgi markers (Golgin-245 or Furin) (Fig. 4A). Arl15-GFP localized predominantly to a Furin- and Golgin-245-positive perinuclear region, as well as to intracellular vesicles. In some cells fluorescence was also detected at the plasma membrane. Brefeldin A (BFA), a fungal inhibitor that interferes with the GTPase cycle of Golgi ARFs and causes the collapse of the Golgi^25–27^, rapidly induced redistribution of Arl15-GFP throughout the cytoplasm, supporting association of Ar1l5 with the Golgi (Supplementary Figure S1). Day-3 3T3-L1 adipocytes showed Arl15-GFP in the perinuclear region, vesicular structures and, albeit with varying relative intensities, the plasma membrane (Fig. 4B). This plasma membrane localisation was particularly visible in some cell lines, including NMuMG (mammary gland epithelial cell line) (Fig. 4C). The presence of Arl15 in these intracellular compartments is consistent with a role for this small G protein in trafficking to the plasma membrane.

Treatment of adipocytes with BFA has been shown to block adiponectin secretion^24,28^ suggesting that this requires an intact Golgi. To investigate if collapse of the Golgi also played a role in inhibition of adiponectin secretion in DOX-treated shArl15 cells (Fig. 1E), we used antibodies against the Golgi marker Syntaxin 6. No evidence of compromise of Golgi structure was seen upon *Arl15* knockdown (Supplementary Figure S2), consistent with lack of effect of *Arl15* knockdown on adipsin secretion (Fig. 1G), which also depends on integrity of the Golgi and post-Golgi trafficking^24^.

To test the possibility that Arl15 acts directly on compartments utilised in adiponectin trafficking we examined co-localization of Arl15-GFP with endogenous adiponectin in day-5 adipocytes using confocal microscopy. Despite the cytoplasm-wide presence of adiponectin-positive vesicular compartments, no extensive overlap (non-thresholded average Pearson’s correlation coefficients (*r*) of 0.39) of the two proteins was observed, however (Fig. 4D).

### Identification of two lipodystrophic patients with ARL15 haploinsufficiency

**Figure 5:**
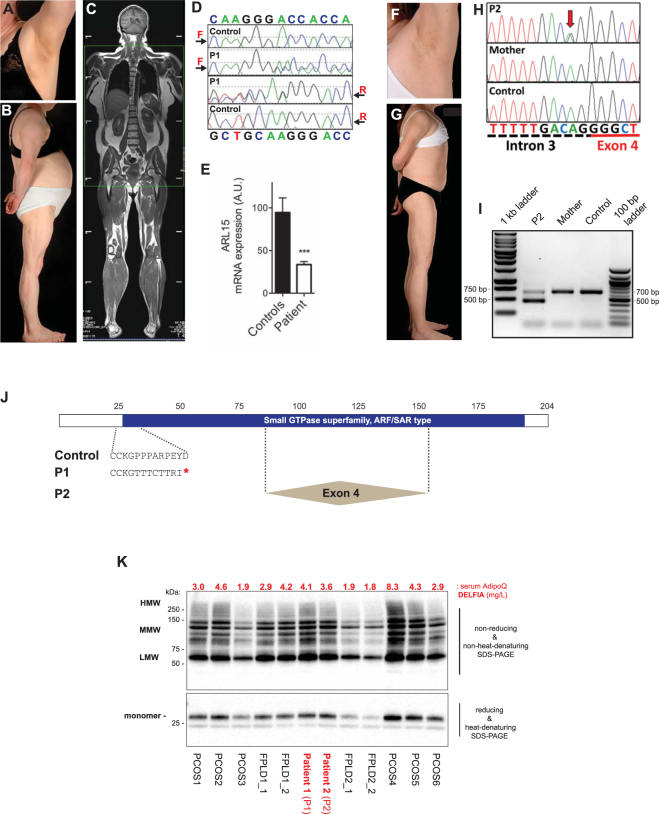
Identification of two lipodystrophic patients with ARL15 haploinsufficiency. (**A**) Axillary acanthosis nigricans in P1 with (**B**) loss of femorogluteal and accumulation of truncal fat. (**C**) Coronal section of whole body MRI of P1 showing femorogluteal lipodystrophy. (**D**) Sequencing chromatograms showing frameshift mutation in *ARL15* in P1 but not a control. (**E**) *ARL15* transcript levels in immortalized lymphoblastoid cells from P1 compared to cells from healthy controls. RT-qPCR data are normalized to *36B4* expression. Means ± SEM are shown (*n* = 3). A.U., arbitrary units. Differences between means were analysed by Student’s *t* test; ***P < 0.001. (**F**) Axillary acanthosis nigricans in P2. (**G**) Femorogluteal lipodystrophy in P2 with mammary hypoplasia. (**H**) Sequencing of *ARL15* in P2, her mother, and a healthy control. The red arrow indicates the heterozygous 3′ splice site mutation in P2. (**I**) Detection of *ARL15* exon 4 (209 bp) in lymphoblastoid cell mRNA. Two PCR products are visible for P2: spliced exon 4-containing and exon 4-less. For P2’s mother and healthy control only the exon 4-containing PCR amplicon is detected. (**J**) Schematic of ARL15 with the predicted Small GTPase superfamily ARF/SAR type functional domain shown in blue. The frameshift caused by the single nucleotide insertion in P1 results in eight aberrant amino acid residues immediately followed by a translation termination codon. The portion of ARL15 deleted in P2 as a result of mis-splicing is indicated by the two dashed lines flanking the box labelled “Exon 4”. (**K**) Immunoblotting analysis of serum adiponectin from the affected patients and controls with either polycystic ovary syndrome (PCOS), idiopathic familial partial lipodystrophy type 1 (FPLD1), or familial partial lipodystrophy type 2 due to *LMNA* p.Arg482Trp heterozygosity. Top, high-molecular weight (HMW), middle-molecular weight (MMW), and low-molecular weight (LMW), indicate the different oligomeric states of serum adiponectin resolved under non-reducing and non-heat denaturing conditions, as indicated. The lower panel shows the monomeric form of adiponectin under heat-denaturing and reducing conditions. Serum adiponectin concentrations as determined by immunoassay (DELFIA) are shown above the blots in red typeface (mg/L). Further information about control samples used is found in Supplementary Table 4.

Identification of rare and deleterious coding variants in a gene at a locus implicated by GWAS in a trait of interest, and demonstration that these variants are associated with a severe form of the relevant trait or disease, provides evidence that the gene concerned is the source of the GWAS signal. We thus sequenced coding exons and flanking sequences of *ARL15* using Sanger sequencing in 189 probands with insulin resistance that was disproportionately severe to their total degree of adiposity, in a further 186 such probands using either exome sequencing (n = 62) or targeted next generation sequencing (n = 124), and 2,479 controls (Supplementary Information). Four variants in *ARL15* were thus identified in the severely insulin resistant cohort (Table 1). Two of these variants were missense variants also identified in the ExAC database with low allele frequencies (0.01–0.02%) but not in the control groups in this study. One variant, p.Cys133Arg, was found in a Bengali woman with late onset generalised lipodystrophy in addition to insulin resistance, and the other, p.Ala110Thr, was found in an obese Italian woman. The scaled Combined Annotation Dependent Depletion Score (32) was 18 for p.Cys133Arg and 27 for p.Ala110Thr. A score of 20 or above indicates that a variant falls in the top 0.1% of all possible variants when ranked by potential deleteriousness. Scaled CADD scores are given for all variants identified in ExAC and controls in Supplementary Information (Table 1, Supplementary Table 3). Neither patient was available for further study. The other variants were an early nonsense mutation, p.Pro26Thrfs*9, and an essential splice-acceptor site mutation in intron 3 (c.254–2 A > G; g.54113409 A > G), both in patients with partial lipodystrophy (Fig. 5). Neither mutation was identified in the ExAC database nor in 2,479 controls sequenced in this study (Supplementary Table 3). Furthermore no other essential splice site or nonsense mutations were identified in the control group in this study, although five patients in the ExAC database (which includes around 20,000 patients ascertained through cardiometabolic traits such as type 2 diabetes) did have heterozygous nonsense variants (p.Glu160*(1 individual), p.Arg30*(1), and p.Tyr92*(3)), among a total of 57 protein-altering variants affecting 187 participants (Supplementary Table 3). No parents were available to study for the first proband (proband 1 (P1)), while the mother of the second proband (proband 2 (P2)) did not carry the splice site mutation. Her father was not available for study.

**Table 1:** Characteristics of four severely insulin resistant patients with *ARL15* variants.

ID	Sex	Age years	Ethnicity	B.M.I. kg/m2	Clinical Diagnoses	ARL15 Variant Detected		
						ARL15 variant	ExAC allele frequency	CADD Score
P1	F	53	White British	30.9	Partial Lipodystrophy Congenital Adrenal Hyperplasia	p.Pro26ThrfsTer9	0	
P2	F	22	White British	20.2	Partial Lipodystrophy Yolk Sac Tumour	c.254-2 A > G (g.54113409 A > G)	0	25
P3	F	24	Bengali	14.9 (16 years old)	Late onset generalised lipodystrophy	p.Cys133Arg	25/120366 (0.00021)	18
P4	F	19	Italian	31.2	Obesity PCOS	p.Ala110Thr	16/120,448 (0.00013)	27

B.M.I. = Body Mass Index; ExAC = Exome Aggregation Consortium^32^. All ancestries are included; CADD Score = Scaled Combined Annotation Dependent Depletion Score^33^ [CADDv1.3 accessed January 2017].

Detailed clinical histories of P1 and P2 are given in the Supplementary Information. In brief, P1, who was heterozygous for the p.Pro26ThrfsTer9 mutation (Fig. 5D,J), is a 53 year old woman who developed obesity with severe clinical features of elevated androgenic hormones at 20 years old, leading to diagnosis of non-classical congenital adrenal hyperplasia due to compound heterozygous mutations in the *CYP21A2* gene, and treatment with a mean daily dose of prednisolone of 7.5 mg per day from 25 years old. Extreme elevation of serum triglyceride was noted at 28 years old, and at 30 years old diabetic ketoacidosis developed and insulin therapy was begun. Subsequent metabolic control remained suboptimal despite daily insulin doses up to 250 units per day, with HbA1c levels between 7 and 10%, and fasting plasma triglyceride concentrations from 190–2,200 mg/dL. At 38 years old peripheral lipodystrophy was recorded. P1’s mother, who had diabetes mellitus, and who was said to have had a similar body habitus, died at 68 years of a myocardial infarction. Her father died at 89 years old of heart failure. Neither were available for study.

On clinical assessment at 52 years old her Body Mass Index (B.M.I.) was 30.9 kg/m^2^ and she had centripetal obesity but peripheral lipodystrophy, with calf hypertrophy and little subcutaneous adipose tissue in the femorogluteal region (Fig. 5A–C, Table 2). Head and neck fat was increased, but there was no easy bruising, thin skin nor muscle wasting. There was persisting axillary acanthosis nigricans and marked clinical hyperandrogenism, with androgenetic alopecia and moderate hirsutism. Fasting biochemical evaluation and imaging revealed metabolic dyslipidemia, fatty liver, raised plasma testosterone but normal cortisol. Adiponectin was mildly low only, and not disproportionate to the overall clinical and biochemical profile (Table 2). Epstein Barr virus-transformed lymphoblastoid cells derived from P1 with the p.Pro26ThrfsTer9 mutation expressed *ARL15* mRNA at less than 50% of the level seen in healthy controls (Fig. 5E), but no mutant allele was seen on sequencing of cDNA, consistent with instability of the mutant mRNA (data not shown).

**Table 2:** Clinical and metabolic profile of Patients P1 and P2 with loss-of-function *ARL15* variants.

	P1	P2	Reference Range
Age, years	53	22	
ARL15 variant	p.Pro26ThrfsTer9	c.254-2 A > G (g.54113409 A > G)	
B.M.I., kg/m^2^	30.9	20.2	19–25
% Fat Mass:			
Arms:	40.2	20.2	35.7–42.9^§^
Legs:	16.4	25.6	38.4–46.0^§^
Trunk:	39.6	46.2	44.1–48.8^§^
Liver triglyceride content, %	24.7 (<5.5)	8 (<5)	^¥^
HbA1c, mmol/mol	60	35	<42
Glucose, mg/dL	94	67	<100
Insulin, pmol/L	76	395	0–60
Free fatty acids, mg/dL	8.5	9.1	8–25
Adiponectin, mg/L	3.5 (2.6–14.9)	4.5 (4.4–17.7)	**
Leptin, μg/L	40.1 (14.9–60.2)	38.1 (2.4–24.4)	**
Triglyceride, mg/dL	399	301	<175
HDL-cholesterol, mg/dL	37	35	>42
LDL-cholesterol, mg/dL	^#^	100	<85
ALT, mU/L	36	133	9–52
Testosterone, ng/dL	98	187	<60
Cortisol, μg/dL	18.6	N/A	5–25
Medication	Metformin Insulin (228U/day) Prednisolone (5 mg/day) Acipimox Ciprofibrate Spironolactone Flutamide	Metformin Thyroxine Fenofibrate Orlistat	

^¥^Method specific references ranges are given in brackets; **sex and B.M.I.-adjusted normal range shown in brackets; ^#^could not be calculated. ^§^Controls were 37 adult female volunteers with BMI >30 kg/m^2^.

P2, who carries an essential splice-acceptor site mutation in intron 3 (c.254–2 A > G; g.54113409 A > G) (Fig. 5H), is a 22 year old woman with subcutaneous lipodystrophy involving limbs and trunk including the mammary region. At 2 years old she was treated for a metastatic primary yolk sac tumour with JEB chemotherapy (carboplatin, etoposide, and bleomycin^29^) followed by surgical resection at 2.5 years old. No local or systemic radiotherapy was used. Progressive obesity was noted from around 4 years old. At 11 years old a predominantly centripetal pattern of adipose deposition was noted, as well as acanthosis nigricans. Marked “metabolic” dyslipidaemia (high serum triglyceride and low HDL cholesterol) and polycystic ovary syndrome, consistent with severe insulin resistance, were recorded shortly afterwards. A marked reduction in body mass index ensued, and on detailed evaluation at 16.5 years old, although there was residual centripetal adiposity, there was striking, relative paucity of adipose tissue from limbs and trunk including the mammary region, where there was breast hypoplasia. Head and neck adipose tissue was preserved, and moderate acanthosis nigricans could be seen in skin folds, but there were no clinical signs of dyslipidaemia, enlarged liver or hyperandrogenism.

On assessment at 22 years old height was 1.63 m and B.M.I. 20.2 kgm^−2^. There was subcutaneous lipodystrophy affecting limbs and trunk including the mammary region, however head and neck adipose tissue was largely spared. Widespread moderate acanthosis nigricans was present in flexural regions **(**Fig. 5F,G **)**. Fasting biochemical evaluation and liver imaging revealed persisting metabolic dyslipidemia, fatty liver, raised plasma testosterone and insulin, and relatively normal adiponectin and leptin (Table 2).

PCR amplification of cDNA derived from whole blood of P2 with the intron 3 splice-acceptor site mutation and her mother showed the presence of a truncated mRNA species which was absent from controls (Fig. 5I). Bidirectional Sanger sequencing of the PCR product showed this to be the result of skipping of exon 4, which is predicted to remove amino acids 85 to 154, including significant parts of the canonical GTP-binding motif in the event of the truncated protein being stable (Fig. 5I,J). Finally we compared plasma adiponectin concentrations and oligomeric profiles among P1, P2 and patients with familial partial lipodystrophy or insulin resistant polycystic ovary syndrome to assess whether P1 and P2 had disproportionately low adiponectin, however both P1 and P2 showed quantitative and qualitative adiponectin profiles sitting within the control group (Fig. 5K **)**.

## Discussion

Several intronic single nucleotide polymorphisms (SNPs) at the *ARL15* locus have been associated with components of the metabolic syndrome and its sequelae including plasma adiponectin, insulin, HDL cholesterol, and triglyceride concentrations, and coronary artery disease. The pattern of these associations, allied to associations with anthropometric traits including body shape and obesity, suggests that SNPs at this locus may primarily influence adipose tissue development or function, and give rise to metabolic traits indirectly by a lipodystrophy-like mechanism. Data from the Genotype-Tissue Expression (GTEx) Project indicate that ARL15 is relatively highly expressed in visceral and subcutaneous adipose tissues, however eQTL data do not show significant association between the GWAS SNPs and *ARL15* expression in the adipose tissue tested. Nevertheless adipose tissue is complex, and whether mature subcutaneous adipose tissue reflects the depot and developmental stage at which the *ARL15* SNPs exert their influence is unclear.

We show that Arl15 expression is upregulated during 3T3-L1 adipocyte differentiation, and that its knockdown impairs 3T3-L1 adipocyte differentiation. We also provide evidence for a cell autonomous role for ARL15 in adiponectin expression and secretion from adipocytes, suggesting that adiponectin concentrations in the blood may be associated with ARL15 SNPs by more than one mechanism. The effects observed are consistent with, but do not prove, the case that the metabolic GWAS signals are mediated through effects on ARL15 itself.

We also report two haploinsufficient individuals with peripheral lipodystrophy, and centripetal obesity with dyslipidemic severe insulin resistance, and two less well studied individuals with severe insulin resistance and rare missense variants in *ARL15*. Neither haploinsufficient patient had adiponectin levels or oligomeric profiles that were distinct from control volunteers with primary lipodystrophy or hyperandrogenemic, insulin resistant PCOS. In both haploinsufficient patients we describe there are cofactors, namely lifelong androgen excess and childhood malignancy treated with systemic chemotherapy, each of which may contribute to adipose tissue damage or remodelling. Moreover, although no frameshift, essential splice site or nonsense mutations were identified in 2,479 controls studied, the ExAC database shows three nonsense mutations in five participants. On the other hand more than 20,000 patients in the ExAC component cohorts were ascertained through cardiometabolic traits potentially related to *ARL15*, so this cannot be construed as a healthy control dataset. Thus, while the human genetic studies we describe do not prove that ARL15 haploinsufficiency gives rise to or predisposes to lipodystrophic insulin resistance, we suggest that this possibility warrants further study.

To date there have been no cellular functions ascribed to ARL15, and given that the term ARL merely indicates that the protein is structurally related to ARFs, no functional information can necessarily be inferred from it^23^. Nevertheless as small G proteins from the Rab and ARF families are critical regulators of vesicle trafficking^21–23,28,30^, we assessed whether ARL15 may play a role in adiponectin routing to the cell surface. We report Arl15 to be localised to the Golgi apparatus, and also to vesicular compartments and to the plasma membrane, which is consistent with this. However although adiponectin is also found in the Golgi, from where it is transported in vesicles to the surface of adipocytes^28^, and although Arl15 knockdown reduced adiponectin secretion, co-localization studies revealed no extensive overlap between the two molecules. Arl15’s action may thus be restricted to a subset of adiponectin transport compartments, including the subset which are destined for secretion. Emerging evidence has revealed a broad range of protein trafficking regulatory mechanisms governed by ARFs, encompassing modulation of signalling inputs, actin dynamics and vesicle formation, organelle structure, recycling of specialized membrane compartments, or lipid composition of compartment membranes^20,24,31^. Whichever mechanism is at play in the case of Arl15, it shows some degree of cargo specificity, as adipsin secretion via the Golgi^24^ and insulin-stimulated Glut4 translocation appear to not have been affected by its knockdown. Unpicking which of these possibilities explains our observations will require substantial further study.

In summary, we have studied the function of Arl15 in a murine preadipocyte cell line, and provide evidence that it is associated with the Golgi apparatus, secretory vesicles and the plasma membrane. Knockdown impairs both preadipocyte differentiation and adiponectin secretion from differentiated adipocytes. We also report two haploinsufficient individuals with partial lipodystrophy, increased centripetal adiposity, and severe insulin resistance and dyslipidemia, although in both cases major additional stressors on adipose function were present. We suggest that a possible role for heterozygous ARL15 loss-of-function variants in pathological adipose remodelling and insulin resistance-related traits merits further study.

## Electronic supplementary material

Supplementary Information
