# Supplementary material for: The metabolic syndrome- associated small G protein ARL15 plays a role in adipocyte differentiation and adiponectin secretion
Source: Sci Rep. 2017 Dec 14;7:17593. doi: 10.1038/s41598-017-17746-8 (PMC5730586; doi:10.1038/s41598-017-17746-8)

The metabolic syndrome-associated small G protein ARL15 plays a role in adipocyte differentiation and adiponectin secretion

Nuno Rocha, Felicity Payne, Isabel Huang-Doran, Alison Sleigh, Katherine Fawcett, Claire Adams, Anna Stears, Vladimir Saudek, Stephen O'Rahilly, Inês Barroso, Robert K. Semple

SUPPLEMENTARY INFORMATION

Supplementary Methods

Severe Insulin Resistance Cohort: All patients had severe insulin resistance, defined as fasting insulin above 150 pmol/L or peak insulin on oral glucose tolerance testing >1,500 pmol/L in nondiabetic patients. In cases of complete insulin deficiency it was defined as an insulin requirement above 3 units kg^{-1} day^{-1}. Most patients had a BMI <30 kg/m^2, but at least 58 patients had BMI >30 kg/m^2. Those with partial β-cell decompensation and clinical features including acanthosis nigricans and those with BMI >30 kg/m^2 were included at the investigators' discretion based on detailed evaluation of clinical and biochemical profiles.

Genomic DNA Sequencing: 189 volunteers with severe insulin resistance and 384 healthy controls were sequenced using Sanger sequencing as described previously[1]. In addition, whole exome sequencing (WES) of genomic DNA was performed as part of the Rare Diseases arm of the UK10K Project [2] on 40 further individuals with severe insulin resistance and 1,928 controls, with 22 individuals with severe insulin resistance later undergoing exome sequencing independently of the UK10K Project. Variants were called in these 22 individuals using the Genome Analysis Toolkit HaplotypeCaller (v3.3-0-537228af)[3]. Putative functional variants in *ARL15* were extracted from all WES data. Raw exome sequence is available from the European Genome-Phenome Archive (https://www.ebi.ac.uk/ega/home). Identifiers are supplied below. Finally, targeted sequencing of *ARL15* was performed on 124 additional individuals with severe insulin resistance and 167 controls, largely drawn from cohorts with neuromuscular (n=117) or thyroid disease (n=48), all studied in parallel as part of the UK10K Project. Targeted re-sequencing analysis has been described previously[4]. These samples were whole-genome amplified (GenomiPhi V2 DNA Amplification Kit; GE Healthcare, Little Chalfont, Buckinghamshire, United Kingdom) using 10ng template prior to pull-down. Sequencing was performed as for the UK10K WES samples using a custom-based targeted Agilent library (ELID S04380110) and HaloPlex Target Enrichment Kit. The bait regions covered 3.8 Mb on the autosomes, using 16,277 intervals and 159.3Kb on the X chromosome, using 691 intervals. Collectively, *ARL15* sequence data was available on 375 severe insulin resistant cases and 2,479 non-severe insulin resistant controls.

Identifiers for Exome Sequences Archived in the European Genome-Phenome Archive (https://www.ebi.ac.uk/ega/home):

EGAS00001000130 (UK10K SIR WES); EGAS00001000129, EGAS00001000101, EGAS00001000131, EGAC00001000024, EGAS00001000121, EGAS00001000109, EGAS00001000112, EGAS00001000117, EGAS00001000225, EGAS00001000713, EGAS00001000242 (UK10K WES non severely insulin resistant participants); EGAS00001000488 (non UK10K SIR WES).

Genetic studies – Assessment of ARL15 exon 4 splicing: For the assessment of *ARL15* exon 4 splicing, RNA was obtained from whole blood using the PAXgene Blood RNA System according to the manufacturer's recommended protocol (PreAnalytiX GmbH, Switzerland). cDNA was prepared from 200ng total RNA by M-MLV reverse transcription (Promega). 50ng cDNA was amplified by PCR using the following cDNA-specific forward and reverse primers flanking the sequence corresponding to exon 4 in *ARL15*: forward primer 5'-AGTATTAAAGCAGTGCCATTCCA-3' and reverse primer 5'-GGAAGAGACATGCGGGGAA-3'. Amplification of the target was evaluated by agarose gel electrophoresis prior to Sanger sequencing.

Plasmid Construction: Murine Arl15 was cloned by PCR from a mouse cDNA library using primers 5'-ATGTCTGATCTCCGGATAACTGAG-3' and 5'-TCACATTCTCACTGCCTCGTG-3' and subcloned into pCR4Blunt-TOPO (Invitrogen). The EGFP-tagged Arl15 expression vector was generated by inserting the coding region of murine *Arl15* into pEGFP-N3 (Clontech) using the EcoRI/KpnI restriction sites. The doxycycline-regulated lentiviral vector used to generate the 3T3-L1 stable cell line overexpressing Arl15-GFP was constructed by inserting the relevant coding sequence into the gateway-based entry vector pEN-Tmcs (ATCC MBA-251, LGC Standards) using the SpeI/XhoI restriction sites and site-specific recombination (Gateway LR Clonase II Enzyme Mix, Invitrogen) with

the lentiviral vector pSLIK-Neo as destination vector (ATCC MBA-236, LGC Standards). The lentiviral vector expressing a microRNA-like short hairpin RNA targeting murine Arl15 (shArl15) was constructed by inserting an annealed oligonucleotide linker (sense: 5'-AGCGACCGCTCAGTACAAGAGATCAATAGTGAAGCCACAGATGTATTGATCTCTTGTACT GAGCGGG-3' and antisense: 5'-GGCACCCGCTCAGTACAAGAGATCAATACATCTGTGGCTTCACTATTGATCTCTTGTACTG AGCGGT-3') into the shuttle vector pEN-TGmiRc3 (Addgene plasmid # 25749) prior to site-specific recombination with pSLIK-Neo. The lentiviral short hairpin RNA used as control and targeting firefly luciferase (shLuc) was obtained from Addgene (plasmid # 25745).

Immunofluorescence microscopy: 3T3-L1 preadipocytes were cultured on 12-mm coverslips and fixed in 3.7% paraformaldehyde for 15 min at room temperature, washed twice with PBS, and permeabilized in 0.5% Triton X-100/PBS with 3% BSA (w/v) for 20 min. Day-3 or day-5 3T3-L1 adipocytes were trypsinized and re-seeded onto poly-D-lysine-coated 12-mm coverslips for 36 hours prior to paraformaldehyde fixation as described above. NMuMG cells expressing GFP-Arl15 were cultured on 12-mm coverslips and fixed in ice-cold ethanol for 10 min. Antibodies were diluted in 0.1% Triton X-100/PBS with 3% BSA. The incubation time was 1 h for primary antibodies and 45 min for fluorophore-conjugated secondary antibodies. Fixed cells were mounted on slide glass using ProLong Gold Antifade mounting medium with DAPI (Molecular Probes). For syntaxin 6 immunostaining studies, shArl15 3T3-L1 fibroblasts were treated with either DOX to induce RNAi or PBS as control, trypsinized, resuspended and combined before allowing cells to re-attach onto glass coverslips for 24 hours. Cells were then fixed, permeabilized, and immunostained as described above using antibodies specific to Syntaxin 6. LipidTOX Deep Red, and Alexa Fluor-coupled secondary antibodies we purchased from Molecular Probes and used according to the manufacturer's instructions. All confocal microscopy images were obtained using a Zeiss LSM510 MetaLaser Scanning Microscope (Carl Zeiss, Jena, Germany) controlled by Zen Microscope and Imaging software package (Carl Zeiss). Pearson's correlation coefficients and Costes automatic thresholds were calculated using the Coloc 2 plugin in ImageJ (https://imagej.net/Coloc_2).

Patient Case History

Patient 1 (P1) Case History: P1 is a 53 year old woman who developed secondary amenorrhoea, progressive hirsutism and androgenetic alopecia at 20 years old, leading to diagnosis of non classical congenital adrenal hyperplasia based on short Synacthen testing and later demonstration of compound heterozygosity for a gene conversion/deletion and the p.Val281Ala mutation in the *CYP21A2* gene. 7.5mg prednisolone was started at 24 years old, and serum testosterone over the ensuing 28 years ranged between 60 and 140 ng/dL.

Obesity developed from around 20 years old, and gross lipemia with fasting plasma triglyceride concentration up to 8,800 mg/dL was noted at 28 years old, leading to introduction of Maxepa and bezafibrate. At 30 years old severe diabetic ketoacidosis developed, with plasma glucose of 630 mg/dL, arterial blood pH of 7.0, and lipemic serum. Multiple daily injections of insulin were begun. Over the subsequent 22 years metabolic control remained suboptimal despite daily insulin doses up to 250 units per day, with HbA1c levels between 7 and 10%, and fasting plasma triglyceride concentrations from 190-2,200 mg/dL. At 38 years old peripheral lipodystrophy was recorded, and acipimox was started.

Patient 2 (P2) Case History: P2 is a 22 year old woman with subcutaneous lipodystrophy involving limbs and trunk including the mammary region. At 2 years old she had been found to have a metastatic primary yolk sac tumour which was treated curatively with 5 doses of JEB chemotherapy (carboplatin, etoposide, and bleomycin[5]) followed by surgical resection at 2.5 years old. No local or systemic radiotherapy was used. Subsequent linear growth was normal but progressive obesity was noted from around 4 years old. At 8 years old height was tracking the 75th centile, and weight was on the 97th centile. At 11 years old atypical adipose topography was noted, with a predominantly centripetal pattern of adipose deposition, as well as acanthosis nigricans. Marked “metabolic” dyslipidaemia (namely high serum triglyceride and low HDL-cholesterol) and polycystic ovary syndrome, consistent with severe insulin resistance were recorded shortly afterwards. These led to introduction of metformin and later orlistat. A marked reduction in body mass index ensued, and on detailed evaluation at 16.5 years old height was 1.60 m, weight 59.5 kg, B.M.I. 23.5 kg/m^2, and waist: hip ratio 0.99 (<0.85). Although there was residual centripetal adiposity, there was striking, relative paucity of adipose tissue from limbs and trunk including the mammary region, where there was breast hypoplasia. Head and neck adipose tissue was preserved, and moderate acanthosis nigricans could be seen in skin folds, but there were no clinical signs of dyslipidaemia, enlarged liver or hyperandrogenism.

Supplementary Tables

Antibody to:	Description	Commercial source
mouse Adipsin	Goat polyclonal (P-16; sc-12402)	Santa Cruz Biotechnology
human Adiponectin	Rabbit polyclonal antibody (ab13881)	Abcam
mouse Adiponectin	Mouse monoclonal (MAB3608)	Millipore
AKT	Rabbit polyclonal (#9272)	Cell Signalling
Phospho-AKT (Ser 473)	Rabbit polyclonal (#9271S)	Cell Signalling
Phospho-AKT (Thr 308)	Rabbit monoclonal (#4056S)	Cell Signalling
Phospho-p42/44 ERK1/2	Mouse monoclonal (#9106S)	Cell Signalling
Syntaxin 6	Mouse monoclonal (Clone 30)	BD Transduction Laboratories
GAPDH	Goat polyclonal (ab9483)	Abcam

Supplementary Table 1: Antibodies

Gene	Species	Forward primer	Reverse primer	Probe
AdipoQ	Mouse	CAGTGGATCTGAC GACACCAA	TGGGCAGGATTAAG AGGAACA	[6FAM]- GGGCTCCAGGATGCTA CTGTTGCAAGC- [TAMRA]
Pparg2	Mouse	GATGCACTGCCTA TGAGCACTT	AGAGGTCCACAGAG CTGATTCC	[6FAM]- AGAGATGCCATTCTGG CCCAC-[TAMRA]
Glut4	Mouse	ACTCATTCTTGGAC GGTTCCTC	CACCCCGAAGATGA GTGGG	[6FAM]- TGGCGCCTACTCAGGG CTAACATCA-[TAMRA]
CypA	Mouse	TTCCTCCTTTCACA GAATTATTCCA	CCCGCCAGTGCCAT TATGG	[6FAM]- ATTCATGTGCCAGGGT GGTGACTTTACAC- [TAMRA]
36B4	Human	GCAGATCCGCATG TCCCTT	TGTTTTCCAGGTGCC CTCG	[6FAM]- AAGCTGTGGTGCTGAT GG-[TAMRA]
**TaqMan Gene Expression Assays used for mRNA quantification**				
**Gene**	**Species**	**ABI Catalog number**		
ARL15	Human	Hs00219491_m1		
Arl15	Mouse	Mm00553694_m1		
aP2	Mouse	Mm00445878_m1		

Supplementary Table 2: Primers and Probes used for mRNA quantification

Protein Consequence	Transcript Consequence	Minor Allele Count (ExAC)	Total Allele Count (ExAC)	Minor Allele Count (This Study)	Total Allele Count (This Study)	CADD score
p.Glu160Ter	c.478G>T	1	26798	0	3856	48
p.Arg30Ter	c.88C>T	3	119454	0	3856	38
p.Tyr92Ter	c.276C>G	1	119978	0	3856	37
p.Arg95Leu	c.284G>T	1	120116	0	3856	35
p.Arg166Cys	c.496C>T	2	29832	1	3856	34
p.Glu154Lys	c.460G>A	1	117952	0	3856	34
p.Arg95Cys	c.283C>T	2	120100	0	3856	34
p.Arg90Trp	c.268C>T	3	119782	0	3856	34
p.Arg150His	c.449G>A	2	118900	0	3856	33
p.Gly84Arg	c.250G>C	1	22470	0	3856	33
p.Phe105Leu	c.313T>C	1	120406	0	3856	32
p.Lys24Asn	c.72G>T	2	119688	0	3856	32
p.Leu127Phe	c.379C>T	2	120458	0	3856	30
p.Leu190Pro	c.569T>C	1	27098	0	3856	30
p.Leu164Pro	c.491T>C	1	28886	0	3856	30
p.Gly102Ala	c.305G>C	1	120308	0	3856	29
p.Leu52Phe	c.154C>T	3	113554	0	3856	28
p.Arg166His	c.497G>A	3	30034	0	3856	28
p.Leu49Phe	c.147G>T	1	114362	0	3856	28
p.Arg150Gly	c.448C>G	1	119096	0	3856	28
p.Ala110Thr	c.328G>A	16	120448	1	3856	27
p.Val61Met	c.181G>A	2	106972	0	3856	27
p.Cys53Phe	c.158G>T	1	112912	0	3856	27
p.Ser94Ile	c.281G>T	1	120062	0	3856	27
p.Arg120Gly	c.358A>G	1	120492	0	3856	27
p.Leu52Val	c.154C>G	4	113554	0	3856	27
p.Thr46Ala	c.136A>G	15	115754	0	3856	26
p.Ala86Thr	c.256G>A	1	119144	2	3856	26
p.Gln144Lys	c.430C>A	2	119864	0	3856	26
p.Ala29Val	c.86C>T	0	--	1	3856	25
p.Ser62Leu	c.185C>T	17	105736	0	3856	25
p.Cys53Arg	c.157T>C	1	113154	0	3856	25
p.Asp58Asn	c.172G>A	20	109498	1	3856	24
p.Val103Leu	c.307G>T	3	120352	2	3856	24
p.Thr41Lys	c.122C>A	1	117422	0	3856	24
p.Asp14Gly	c.41A>G	1	88378	0	3856	24
p.His143Tyr	c.427C>T	1	119970	0	3856	23
p.Arg95His	c.284G>A	1	120116	0	3856	23

p.Ala20Glu	c.58C>A	0	--	1	3856	23
p.Arg5Gln	c.14G>A	1	83234	0	3856	23
p.Val61Leu	c.181G>T	1	106972	0	3856	22
p.Val152Ile	c.454G>A	1	118554	0	3856	20
p.Ile89Val	c.265A>G	1	119696	0	3856	19
p.Ala9Gly	c.26C>G	1	89812	0	3856	19
p.Cys133Arg	c.397T>C	25	120366	1	3856	18
p.Ala9Val	c.26C>T	1	89812	0	3856	16
p.Ala9Glu	c.26C>A	1	89812	0	3856	16
p.Lys51Arg	c.152A>G	1	113858	0	3856	14
p.Leu35Val	c.103C>G	1	118906	0	3856	8
p.Val60Ile	c.178G>A	14	108178	0	3856	8
p.Asp181Glu	c.543T>G	15	29108	1	3856	0
p.Asp58Glu	c.174T>A	3	109332	0	3856	0

Supplementary Table 3: *ARL15* variants altering protein sequence identified in control populations. All variants in the ExAC database (accessed January 2017) or identified in this study in *ARL15* are shown. Nonsense mutations are highlighted in red, but no essential splice site mutations were found. Two variants also found in P3 and P4 in the severe insulin resistance cohort are highlighted in green. Variants are ordered according to CADD scores, which are shown rounded to the nearest whole number. A total of 25 individuals with heterozygous *ARL15* variants with a CADD score of 30 or higher were identified, and 132 individuals with heterozygous *ARL15* variants with a CADD score of 20 or higher. The mean number of alleles studied for all ExAC variants was 105,271.

ID	Age, years	Sex	B.M.I., kg/m^2	Clinical Diagnosis	Serum adiponectin concentration, mg/L (**)	Fasting plasma insulin, pmol/L (NR 0-60)	Fasting blood glucose, mg/dL (NR 3.6-5.5)
PCOS1	43	F	40.0	PCOS	3.0	101	9.5
PCOS2	46	F	44.8	PCOS	4.6	74	5.5
PCOS3	43	F	32.7	PCOS	1.9	247	4.5
FPLD1_1	45	F	38.9	FPLD1	2.9	150	11
FPLD1_2	50	F	28.5	FPLD1	4.2	71	5.9
FPLD2_1	25	F	28.3	LMNA	1.9	50	7.3
FPLD2_2	27	F	25.2	LMNA	1.8	341	5.6
PCOS4	20	F	33.3	PCOS	8.3	103	4.4
PCOS5	17	F	39.6	PCOS	4.3	87	5.0
PCOS6	16	F	29.0	PCOS	2.9	259	4.9

Supplementary Table 4: Control samples used for serum adiponectin blotting shown in Figure 5. B.M.I. = Body Mass Index; NR = Normal Range; PCOS = polycystic ovary syndrome; FPLD1 = Idiopathic familial partial lipodystrophy type 1; FPLD2 = Familial partial lipodystrophy type 2, due in both cases to the heterozygous *LMNA* p.Arg482Trp mutation. ** NR for adiponectin are BMI-specific: for BMI <25 kg/m^2, adiponectin NR = 4.4-17.7; for BMI 25-30 NR = 3.5-15.5; for BMI 30-35 NR = 2.6-14.9.

Supplementary Figures

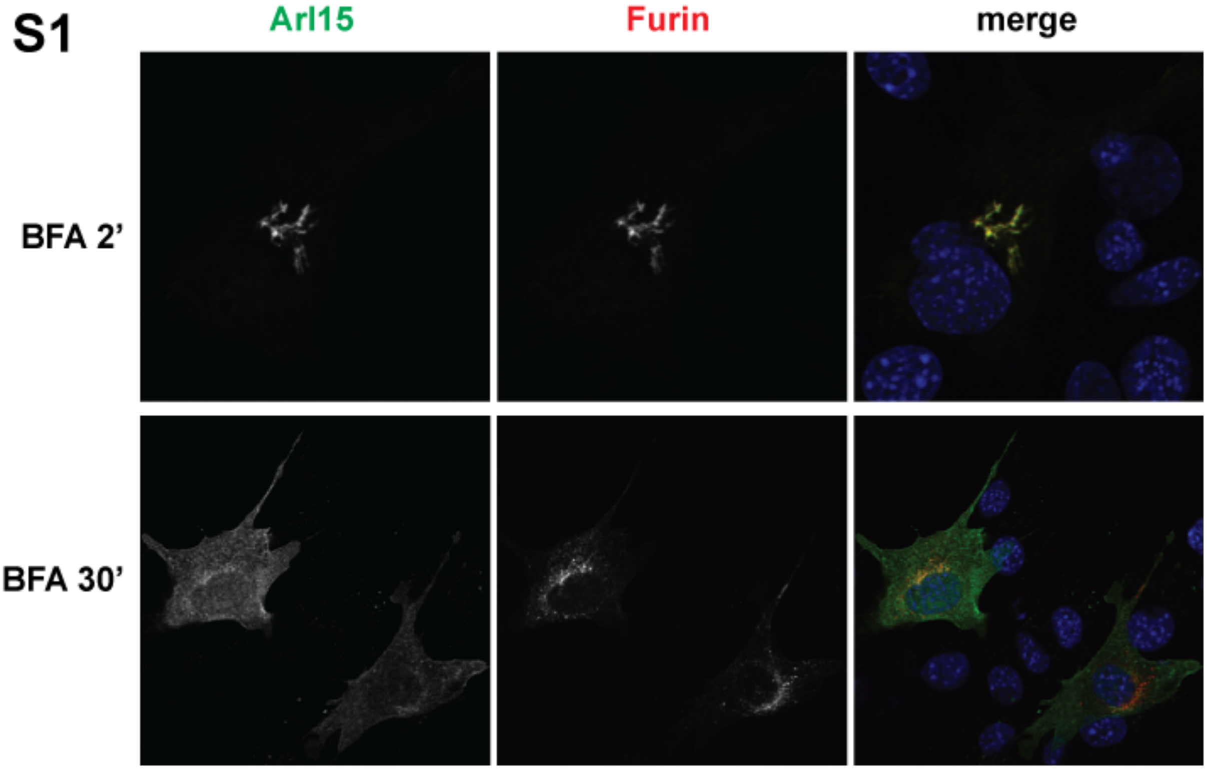

Supplementary Figure S1: Brefeldin A (BFA) induces re-distribution of Arl15-GFP. 3T3-L1 preadipocytes co-expressing Arl15-GFP and CFP-Furin (a Golgi marker) were pre-treated with BFA for 2 min or 30 min, as indicated, fixed, and imaged by confocal fluorescence microscopy. Pre-treatment with BFA for 2 min caused mild perturbation of Golgi morphology and Arl15-GFP distribution (for representative images of non-treated cells refer to Figure 4A). 30 min of BFA treatment resulted in the collapse of the Golgi apparatus and redistribution of Arl15-GFP throughout the cytoplasm.

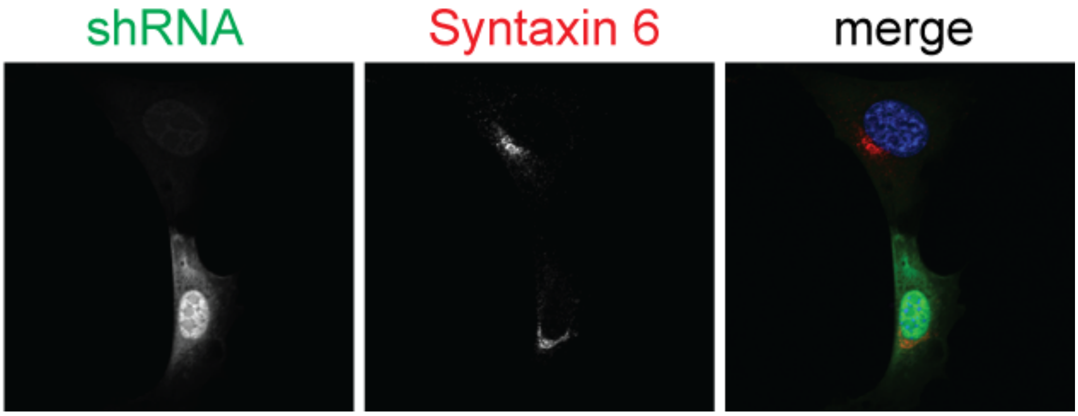

Supplementary Figure S2: Golgi integrity is maintained upon RNAi-mediated knockdown of Arl15. Independent cultures of shArl15 3T3-L1 preadipocytes were treated with DOX or PBS, trypsinized and pooled prior to fixation and immunostaining with antibodies specific to Syntaxin 6 (a Golgi marker) and imaging by confocal microscopy. DOX-induced expression of the microRNA-like shRNA targeting *Arl15* is coupled to the expression of a heterologous mRNA encoding GFP through a monocistronic transcript. The cell at the bottom of the micrograph has been treated with DOX and is therefore expressing both FP and an shRNA against *Arl15*. The cell at the top has been treated with PBS only, as negative control. The Golgi apparatus remained intact upon induction of RNAi.

Supplementary Information References

1. Fawcett, K. A. *et al.* Evaluating the role of LPIN1 variation in insulin resistance, body weight, and human lipodystrophy in U.K. populations. *Diabetes* **57,** 2527–2533 (2008).
2. UK10K Consortium *et al.* The UK10K project identifies rare variants in health and disease. *Nature* **526,** 82–90 (2015).
3. McKenna, A. *et al.* The Genome Analysis Toolkit: a MapReduce framework for analyzing next-generation DNA sequencing data. *Genome Res.* **20,** 1297–303 (2010).
4. Grozeva, D. *et al.* Targeted Next-Generation Sequencing Analysis of 1,000 Individuals with Intellectual Disability. *Hum. Mutat.* **36,** 1197–1204 (2015).
5. Pinkerton, C. R. *et al.* 'JEB'--a carboplatin based regimen for malignant germ cell tumours in children. *Br. J. Cancer* **62,** 257–62 (1990).

Full-length blots:

From Figure 1:

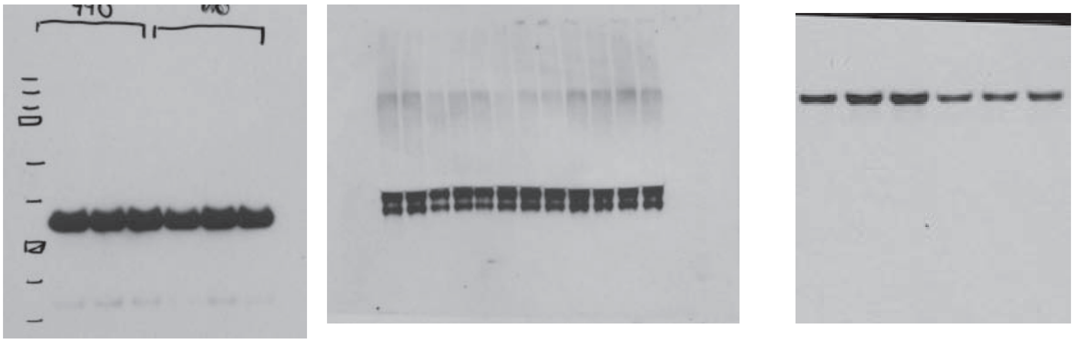

From Figure 2:

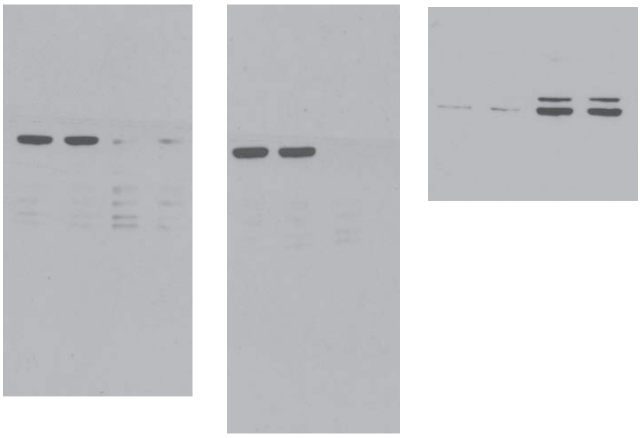

From Figure 5:

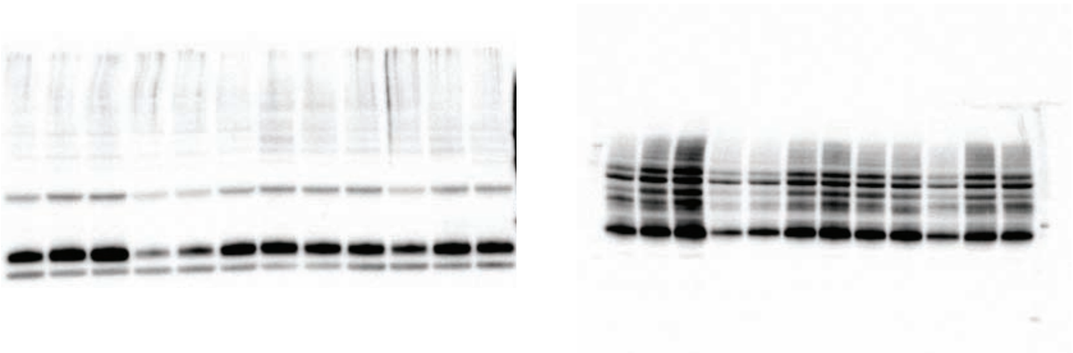

Supplement: Supplementary file 1 — Supplementary Information [file 41598_2017_17746_MOESM1_ESM.pdf]
